# Associations between various types of activity and physical frailty in older Japanese: a cross-sectional study

**DOI:** 10.1186/s12877-023-04501-0

**Published:** 2023-11-29

**Authors:** Tsubasa Yokote, Harukaze Yatsugi, Tianshu Chu, Xin Liu, Hiro Kishimoto

**Affiliations:** 1 Department of Behavior and Health Sciences, Graduate School of Human-Environment Studies, Kyushu University, Fukuoka, 819-0395 Japan; 2 Faculty of Arts and Science, Kyushu University, 744 Motooka Nishi-ku, Fukuoka, 819-0395 Japan; 3 Epidemiological Study Group, Medical Evidence Division, Intage Healthcare Inc., Tokyo, 101-0062 Japan; 4 Center for Health Science and Counseling, Kyushu University, Fukuoka, 819-0395 Japan

**Keywords:** Exercise, Social participation, Physical activity, MVPA, Tri-axis accelerometer, Frailty, Cross-sectional study, Older adults, Japanese, Itoshima Felix Study

## Abstract

**Background:**

Physical activity is known to help prevent physical frailty, but it is not clear which physical activities practiced alone or in combination are most closely associated with a lower risk of physical frailty. We investigated differences in the associations of exercise habit, social participation, and moderate-to-vigorous physical activity (MVPA) with physical frailty and its components among community-dwelling older Japanese adults.

**Subjects and methods:**

A total of 831 older adults participated in this cross-sectional study. Physical frailty was defined based on the Fried et al. criteria. Exercise habit was defined as exercising ≥ 30 min per day for ≥ 2 days per week for ≥ 1 year. Social participation was defined as participating in community activities ≥ 1/week. MVPA was defined as ≥ 300 min/week of moderate physical activity (MPA) or 150 min/week of vigorous physical activity (VPA). We classified the participants into eight groups according to the presence/absence of these activities, and we performed a logistic regression analysis to investigate the association between different activities, both alone and in combination, and physical frailty.

**Results:**

The prevalence of physical pre-frailty + frailty was 74.8% in the None group, 65.0% in the Exercise habit group, 76.3% in the Social participation group, 56.5% in the MVPA group, 58.7% in the Exercise habit + Social participation group, 44.0% in the Exercise habit + MVPA group, 41.3% in the Social participation + MVPA group, and 38.0% in the All group. Compared to the None group, the groups in which participants were engaged in any combination of two or more types of activity, as well as the MVPA-alone group had significantly lower risks of physical pre-frailty + frailty.

**Conclusion:**

Community-dwelling older Japanese adults who engaged in MVPA or any combination of two or more types of physical activity as defined herein had lower risks of physical pre-frailty and frailty.

**Supplementary Information:**

The online version contains supplementary material available at 10.1186/s12877-023-04501-0.

## Introduction

One of the geriatric syndromes is frailty, a condition of vulnerability to stress due to decreased physiological reserves in old age [1]. Physical frailty is a concept that is a precursor to the state of needing long-term care [2]. Japanese have the world’s highest healthy life expectancy [3]. However, the prevalence of frailty has been reported to increase with age. There is concern about the increasing older individuals with frailty. Frailty has physical, psychiatric, and social aspects, and among these, physical frailty is directly related to the need for nursing care and the risk of disease [4, 5]. It is thus necessary to identify factors that are associated with a low risk of physical frailty in community-dwelling older adults as well as factors that can be modified to help older adults avoid becoming frail. The finding of the association between physical frailty and its risk or prospective factors in Japan could provide important information worldwide.

A review and network meta-analysis of various intervention studies (67 randomized controlled trials) of frailty prevention in older adults demonstrated that among various factors, physical activity is the most effective in preventing frailty [6]. However, the question of what exercises or activities are the most effective in terms of the content of physical activity has remained. Our early research showed that compared to a group of subjects who had no exercise habit, subjects with a high-frequency or long-duration exercise habit had a lower risk of physical frailty [7]. It has also been reported that the risk of physical frailty is cross-sectionally lower with more social participation (other than social exercise participation) and with moderate-to-vigorous physical activity (MVPA) [8, 9]. However, it is not clear what type(s) of physical activities alone or in combination are associated with a lower risk of physical frailty. We conducted the present study to investigate the differences in the associations between physical frailty and (*i*) exercise habit, (*ii*) social participation, and (*iii*) MVPA, each alone and in combination, among community-dwelling older adults.

## Participants and methods

### Study design and participants

This was a cross-sectional study using baseline data from the Itoshima Felix Study conducted in 2017 [10]. The participants (aged 65–75 years) lived in Itoshima City, Fukuoka Prefecture, Japan and were not certified for support or nursing care in the Long-Term Care Insurance System [11].

The Community Needs Survey (CNS) led by the Japanese government has been used to examine residents’ health status, living conditions, service usage, utilization of local resources, satisfaction with community life, and demands. We set the population for our study as the 10,000 citizens aged 65–75 years who responded to the CNS in 2016. Considering the size of the district, we randomly selected 5,000 individuals and mailed them an invitation to complete the CNS and our questionnaires. Of these individuals, 930 completed physical function tests and additional questionnaires at their local community centers. A final total of 831 participants (388 men and 443 women) who met the criteria for valid data on physical activity measured by a triaxial accelerometer and had no missing data on physical frailty were included in the final analysis. There were no participants with missing data on exercise habit or social participation.

The accelerometer (Active Style Pro HJA-350IT, Omron Healthcare, Kyoto, Japan) was worn for 7 consecutive days, and we defined ‘valid data’ as the data from the accelerometer worn consistently for ≥ 10 h/day on ≥ 4 days [12–14]. The accelerometer data are known to be more accurate than estimates from self-reported questionnaires, and the use of such accelerometers is increasing in general populations [15]. The accuracy of the intensity estimated by the Active Style Pro accelerometer has been validated with the Douglas bag method [16]. The use of a triaxial accelerometer to assess physical activity also allowed for a more accurate estimate of activity intensity compared to that of a conventional uniaxial accelerometer [16].

### Measurement of frailty status

The criteria of physical frailty were determined based on the frailty phenotype, with values in the bottom 20% of the population [1, 17]. These criteria consist of five items: shrinking, weakness, exhaustion, slowness, and low physical activity. In this study we defined physical ‘frailty’ as meeting three or more of the five criteria; we defined physical ‘pre-frailty’ as meeting one or two of the criteria, and ‘robust’ status as meeting none of the criteria. Shrinking was defined as responding “yes” to the question, “Have you experienced unintentional weight loss > 2–3 kg in the previous six months?“.

Exhaustion was assessed by extracting two items from the six-item method of assessing psychological stress developed by Kessler et al. [18]. The participants were asked how they felt in the last month: “Did you feel that everything you did was an effort?“ and “Did you feel exhausted without any reason?“ with five response options: ‘not at all,‘ ‘a little,‘ ‘sometimes,‘ ‘mostly,‘ and ‘always.‘ If the answer to any of the questions was ‘sometimes,‘ ‘mostly,‘ or ‘always,‘ the respondent was considered to be exhausted.

Weakness was defined as scoring in the lowest 20% of maximum grip strength and was stratified by gender and body mass index (BMI). Grip strength was measured using a Smedley grip strength meter (GRIP-D, T.K.K. 5401; Takei Scientific Instruments, Niigata, Japan) with the subject’s arms directly down while he or she was in an upright position. The measurement was taken for each hand, alternating between hands, and repeated. We averaged the greater values for both hands [19].

Slowness was defined as being included in the bottom 20% of maximum walking speed based on the 5-meter walking test, stratified by gender and height. For the gait speed measurement, an 11-m walking path was created and the participants were instructed to walk the 11-m distance from a stationary standing position, and the time was measured at the 5-m distance between 3-m and 8-m points [20]. Low physical activity was evaluated by measuring the participant’s physical activity energy expenditure (PAEE, kcal) using the above-mentioned triaxial accelerometer. Low physical activity was defined as scoring in the lowest 20% of energy expenditure of physical activity per day, stratified by gender. The results were quantified as kilocalories per kilogram of body weight expended per day (kcal/kg/day).

### Measurement of exposure factors

Three exposure factors were measured: the presence and degree of an exercise habit, the participant’s social participation, and MVPA. Exercise habit is defined by the Ministry of Health, Labor and Welfare in Japan as exercising for ≥ 30 min/day on ≥ 2 days/week for ≥ 1 year, which is commonly used [21]. The exercise items were as follows: Japanese *sampo* (i.e., strolling, including dog walking), walking, hiking, cycling, gymnastics (including stretching), yoga, golf, golf swing, ground golf, table tennis, ballroom dancing, aerobic dance, tai chi, underwater exercise, volleyball, catch ball, bowling, strength training, and others. Social participation was defined as participating in community activities such as festivals, neighborhood associations, club activities, volunteer activities, religious activities, and commerce and industry associations ≥ 1/week. Frequency was defined as described in a large Asian cohort study [22].

MVPA: The World Health Organization recommends 150–300 min of moderate physical activity (MPA: activity intensity of 3.0 METs to 5.9 METs) or 75–150 min of vigorous physical activity (VPA: activity intensity ≥ 6.0 METs) per week for older adults to prevent various diseases, and for more benefit, > 300 min of MPA or > 150 min of VPA per week is recommended [23]. Their MPA and VPA levels were measured with the aforementioned triaxial accelerometer, and those who engaged in either ≥ 300 min MPA/week or ≥ 150 min VPA/week were then included and defined as those who met the MVPA criterion.

### Other measurements on potential confounders

We obtained the following information from each participant via questionnaire: age, gender, BMI, medical history (presence/absence of osteoporosis, hypertension, dyslipidemia, diabetes, cerebral infarction, cardiac disease, and other diseases), number of pain sites (presence/absence of each shoulder, elbow, wrist, hip, knee, ankle, waist, and neck), years of education, tobacco smoking habit (selected from ‘almost every day,‘ ‘sometimes,‘ ‘used to smoke but quit,‘ and ‘never’), and alcohol consumption (selected from ‘almost every day,‘ ‘sometimes,‘ ‘rarely,‘ and ‘never’). The participants’ cognitive function was measured with the Mini-Mental State Examination (MMSE). Sedentary time (activity intensity < 1.5 METs) was measured with the above-mentioned triaxial accelerometer.

### Data analysis

The comparison of characteristics by the presence or absence of exercise habit was performed with the χ^2^ test, t-test, or Wilcoxon rank sum test. We conducted ordinal logistic regression analyses to examine the associations between the three activities (exercise habit, social participation, MVPA) and frailty status (frailty/pre-frailty/robust). On the other hand, a binomial logistic regression analysis was performed to examine the association of exercise habits, social participation, and MVPA alone, or their combinations, with physical pre-frailty and frailty. Due to the small number of individuals with physical frailty, the outcome of this analysis was categorized into two binary groups: physical pre-frailty or frailty, and robust. The participants who were not engaged in any of the activities were designated as the ‘None group’, while those who were engaged only in exercise habit, social participation, or MVPA were designated as the ‘Exercise habit group’, ‘Social participation group’, or ‘MVPA group’, respectively. The combination groups are the ‘Exercise habit + Social participation group’, the ‘Exercise habit + MVPA group’, the ‘Social participation + MVPA group’, and the ‘All group’. We calculated the odds ratio (OR), 95% confidence interval (95%CI), and *p*-value for both physical pre-frailty + frailty for each group, with the None group as the reference.

Since the association between MVPA and low activity (a component of physical frailty) is likely to be strong, four of the five components of physical frailty (low activity is excluded) are considered the criteria of the ‘four-item frailty phenotype’. We thus also conducted analyses using the following definitions: (*i*) frailty for ≥ 3 of the components of four-item frailty, (*ii*) four-item pre-frailty for one or two components, and (*iii*) four-item robust for none of the four components.

The adjustment factors were age, gender, BMI, number of medical conditions, number of pain sites, MMSE score, years of education, presence/absence of smoking habit (presence of a smoking habit was defined as ‘almost every day’ or ‘sometimes’), presence/absence of alcohol habit (presence was defined as ‘almost every day’ or ‘sometimes’), and sedentary time. Statistical analyses were conducted using Statistical Analysis Software (SAS) ver. 9.4 (SAS, Cary, NC, USA). The computations were carried out using the computer resources offered under the General Projects category of the Research Institute for Information Technology, Kyushu University. The significance level was set at p < 0.05.

## Results

The basic characteristics of the participant groups classified according to the activities they were engaged in are shown in Table 1. The prevalence of physical pre-frailty + frailty tended to be lower in the MVPA-alone group and in the two-or-more types of activity groups.

**Table 1:** Characteristics of the older community-dwelling Japanese adults and their combinations of physical activity

	None n = 139	Exercise habit n = 60	Social participation n = 76	MVPA n = 115	Exercise habit + Social participation n = 104	Exercise habit + MVPA n = 116	Social participation + MVPA n = 63	All activities n = 158
Age, yrs	71 [68–74]	72 [69–74.0]	70 [68–74]	70 [68–71]	72 [69–74]	71 [68–74]	69 [67–72]	70 [68–73]
Men	67 (48.2)	36 (60.0)	37 (48.7)	43 (37.4)	47 (45.2)	62 (53.5)	19 (30.2)	77 (48.7)
BMI, kg/m^2^	23.2 ± 3.4	23.2 ± 2.9	23.2 ± 3.1	22.6 ± 3.3	23.2 ± 3.1	22.6 ± 3.1	23.0 ± 3.6	22.4 ± 2.8
Presence of disease	122 (87.8)	51 (85.0)	63 (82.9)	83 (72.2)	82 (78.9)	88 (75.9)	42 (66.7)	114 (72.2)
No. of pain sites	1 [0–3]	1 [0–2]	2 [1–3]	1 [0–3]	1 [0–3]	1 [0–2]	1 [0–2]	1 [0–2]
Education, yrs	12.8 ± 2.3	13.0 ± 2.7	13.2 ± 2.1	12.5 ± 2.1	13.3 ± 2.5	12.8 ± 2.6	12.8 ± 2.0	13.0 ± 2.4
Presence of smoking habit	16 (11.5)	5 (8.3)	9 (11.8)	9 (7.8)	12 (11.5)	7 (6.0)	3 (4.8)	1 (0.6)
Presence of alcohol habit	64 (46.0)	31 (51.7)	35 (46.1)	60 (52.2)	49 (47.1)	58 (50.0)	23 (36.5)	96 (60.8)
MMSE, score	28 [26–30]	28 [27–30]	29 [26–30]	29 [26–30]	28 [26.0–30.0]	28 [26–30]	29 [27–30]	28 [26–30]
Sedentary time, min/day	480.5 ± 108.9	492.9 ± 102.2	484.4 ± 93.7	392.3 ± 116.7	482.2 ± 89.5	433.1 ± 105.3	425.4 ± 89.2	408.0 ± 97.5
Physical pre-frailty	87 (62.6)	34 (56.7)	51 (67.1)	62 (53.9)	56 (53.9)	51 (44.0)	25 (39.7)	60 (38.0)
Physical frailty	17 (12.2)	5 (8.3)	7 (9.2)	3 (2.6)	5 (4.8)	0 (0)	1 (1.6)	0 (0)

Data are mean ± standard deviation, median [25–75% tiles], or n (%). BMI: body mass index, MMSE: Mini-Mental State Examination

### Associations of exercise habit, social participation, and MVPA with physical pre-frailty and frailty

The associations of exercise habit, social participation, and MVPA with physical pre-frailty and frailty, respectively, are summarized in Table 2. With respect to any of exercise habits, social participation, and MVPA, the proportions of both pre-frailty and frailty were lower for those with participation than without. The univariate ordinal logistic regression analysis results showed that the participants with an exercise habit (OR 0.49, 95%CI: 0.37–0.65), social participation (0.68, 0.52–0.88), and MVPA (0.33, 0.25–0.44) had significantly lower crude ORs of more severe frailty. After the adjustment for confounding factors, the participants with an exercise habit (0.58, 0.43–0.79), social participation (0.72, 0.53–0.96), and MVPA (0.40, 0.30–0.55) had significantly lower adjusted ORs for more severe physical frailty compared to the participants without them, respectively. The OR was the lowest for MVPA group, followed by exercise habit and social participation groups.

**Table 2:** Associations between exercise habits, social participation, moderate-to-vigorous physical activity and more severe frailty: results of the ordinal logistic regression analyses

	n (%)	Physical pre-frailty and frailty, n (%)	Model 1			Model 2		
			OR	95%CI	*p*-value	OR	95%CI	*p*-value
Those without exercise habit	393 (47.3)	Pre-frailty, 225 (57.3) Frailty, 28 (7.1)	1.00	ref.	−	1.00	ref.	−
Those with exercise habit	438 (52.7)	Pre-frailty, 201 (45.9) Frailty, 10 (2.3)	0.49	0.37–0.65	< 0.0001	0.58	0.43–0.79	0.0004
Those without social participation	430 (51.7)	Pre-frailty, 234 (54.4) Frailty, 25 (5.8)	1.00	ref.	−	1.00	ref.	−
Those with social participation	401 (48.3)	Pre-frailty, 192 (47.9) Frailty, 13 (3.2)	0.68	0.52–0.88	0.0043	0.72	0.53–0.96	0.03
Those without MVPA	379 (45.6)	Pre-frailty, 228 (60.2) Frailty, 34 (9.0)	1.00	ref.	−	1.00	ref.	−
Those with MVPA	452 (54.4)	Pre-frailty, 198 (43.8) Frailty, 4 (0.9)	0.33	0.25–0.44	< 0.0001	0.40	0.30–0.55	< 0.0001

Model 1: No adjustment factor. Model 2: Adjusted for age, gender, BMI, number of diseases, number of pain sites, MMSE score, smoking habit, alcohol habit, years of education, sedentary time, presence /absence exercise habit, social participation, and MVPA (moderate-to-vigorous physical activity)

### Associations of exercise habit, social participation, and MVPA alone and in combination with physical pre-frailty + frailty

**Fig. 1:**
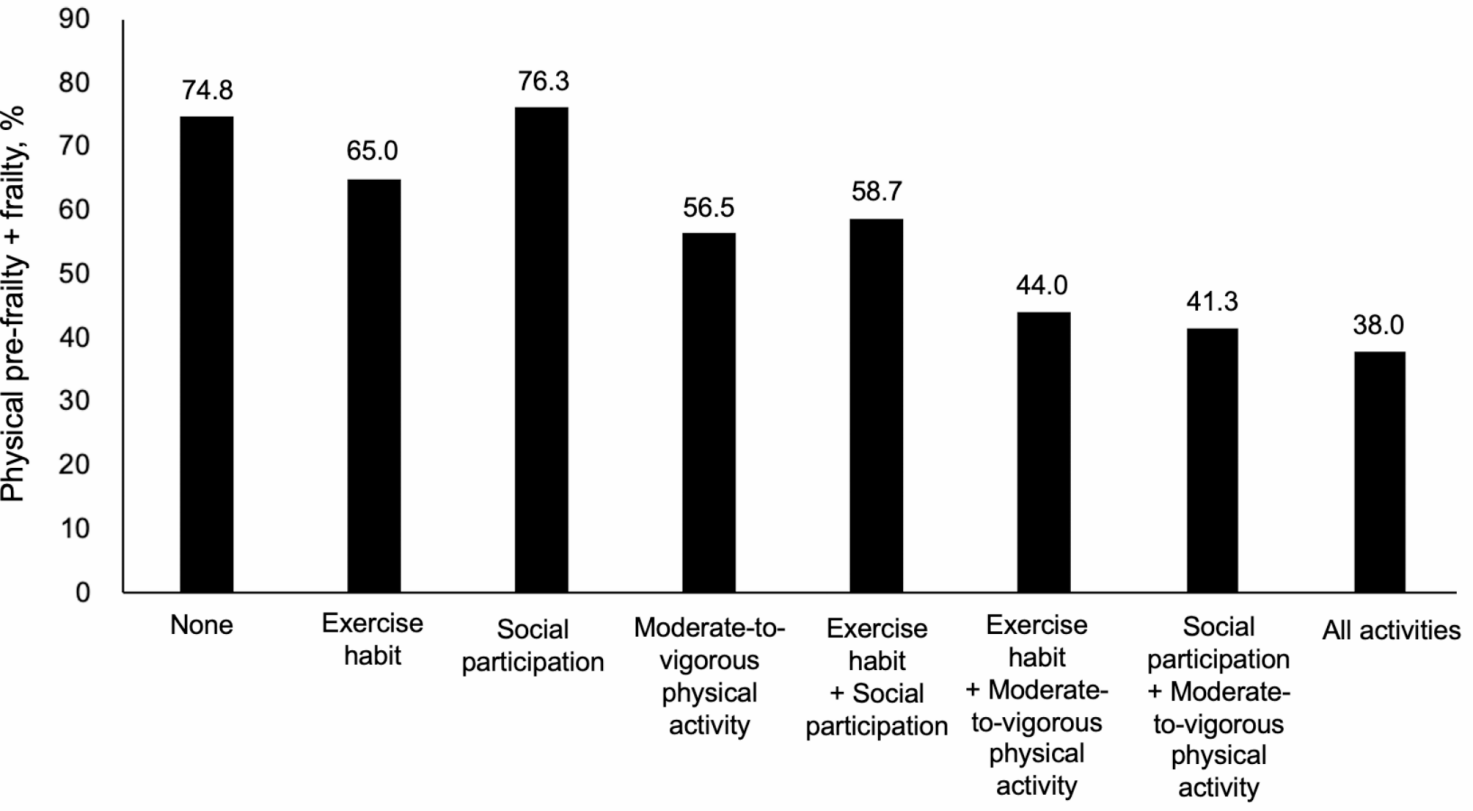
The rate of physical pre-frailty + frailty in each activity group. Exercise habit was defined as exercising for ≥ 30 min/day on ≥ 2 days/week for ≥ 1 year. Social participation was defined as participating in community activities such as festivals, neighborhood associations, club activities, volunteer activities, religious activities, and commerce and industry associations ≥ 1×/week. Those who engaged in either moderate physical activity at ≥ 300 min or vigorous physical activity ≥ 150 min were then included and defined as those who met the moderate-to-vigorous physical activity (MVPA) criterion. The participants who did not practice any of the activities were designated the None group; those who only had an exercise habit were the Exercise habit group, those who only engaged in social participation were the Social participation group, and those who met only the MVPA criterion were the MVPA group. Among these community-dwelling older adults, those with MVPA alone or any combination of two or more activities tended to have lower rates of physical pre-frailty + frailty

Figure 1; Table 3 depict the associations of exercise habit, social participation, and MVPA (alone and in combination) with physical pre-frailty + frailty. The eight categories were classified based on the presence or absence of an exercise habit, social participation, and MVPA, respectively. The rates of physical pre-frailty + frailty were lower in the MVPA-alone group and in the groups combining two or more activities. The association between participation to the particular activities and physical pre-frailty + frailty was analyzed by binomial logistic regression analyses setting the None group as a reference. After multivariable adjustment, the ORs for physical pre-frailty + frailty of the Exercise habit (0.65, 0.33–1.29) or the Social participation (1.10, 0.56–2.18) groups did not differ significantly to that of the None group. The MVPA group had a significantly lower OR for physical pre-frailty + frailty (0.52, 0.29–0.91) compared to the None group. Compared to the None group, all groups with a combination of two or more activities had significantly lower ORs for physical pre-frailty + frailty.

**Table 3:** Association of each activity combination with physical pre-frailty + frailty: results of the binominal logistic regression analyses

Group	n (%)	Pre-frailty + frailty, n (%)	Model 1			Model 2		
			OR	95%CI	*p*-value	OR	95%CI	*p*-value
None	139 (16.7)	104 (74.8)	1.00	ref.	−	1.00	ref.	−
Exercise habit	60 (7.2)	39 (65.0)	0.63	0.33–1.20	0.16	0.65	0.33–1.29	0.22
Social participation	76 (9.2)	58 (76.3)	1.08	0.56–2.08	0.81	1.10	0.56–2.18	0.77
MVPA	115 (13.8)	65 (56.5)	0.44	0.26–0.74	0.0023	0.52	0.29–0.91	0.02
Exercise habit + Social participation	104 (12.5)	61 (58.7)	0.48	0.28–0.83	0.008	0.44	0.25–0.77	0.0046
Exercise habit + MVPA	116 (14.0)	51 (44.0)	0.26	0.16–0.45	< 0.0001	0.29	0.16–0.50	< 0.0001
Social participation + MVPA	63 (7.6)	26 (41.3)	0.24	0.13–0.45	< 0.0001	0.27	0.14–0.53	0.0001
All activities	158 (19.0)	60 (38.0)	0.21	0.13–0.34	< 0.0001	0.22	0.13–0.37	< 0.0001

Model 1: No adjustment factors. Model 2: Adjusted for age, gender, BMI, no. of diseases, no. of pain sites, MMSE score, smoking habit, alcohol habit, years of education, and sedentary time

The ORs were the lowest for the All group (0.22, 0.13–0.37), followed by the Social participation + MVPA group (0.27, 0.14–0.53), the Exercise habit + MVPA group (0.29, 0.16–0.50), the Exercise habit + Social participation group (0.44, 0.25–0.77) and the MVPA-alone group.

### Associations of exercise habit, social participation, and MVPA alone and in combination with the components of physical frailty

The associations of exercise habit, social participation, and MVPA alone and in combination with the components of physical frailty are described in Additional File 1. There were no components of physical frailty in the Exercise habit group or Social participation group that showed a significantly lower OR compared to the None group. The following results were observed compared to the None group. The MVPA group had a significantly lower OR for low physical activity (0.02, 0.003–0.17). The Exercise habit + Social participation group had a significantly lower OR for slow gait speed group (0.47, 0.25–0.88). The Exercise habit + MVPA group had significantly lower ORs for slow gait speed (0.30, 0.14–0.61) and low physical activity (0.09, 0.03–0.26). The MVPA + Social participation had significantly lower ORs for slow gait speed (0.28, 0.11–0.72) and low physical activity (0.06, 0.01–0.29), and the All group had significantly lower ORs for slow gait speed (0.29, 0.15–0.57), exhaustion (0.32, 0.15–0.69), and low physical activity (0.05, 0.01–0.18).

### Analysis of the components of frailty excluding low physical activity

In the analysis excluding low physical activity from the components of physical frailty, the association between the MVPA group and the four-item pre-frailty/frailty was not statistically significant, unlike before excluding low physical activity (Additional File 2).

## Discussion

The community-dwelling older Japanese adults who engaged in an exercise habit, social participation, or MVPA as defined in this study were all at lower risk of more severe physical frailty compared to those without these respective activities. However, regarding each of these three activities alone, only the MVPA group had a significantly lower risk of low physical activity in the physical frailty component and for physical pre-frailty and frailty. The risk of physical pre-frailty and frailty was low for all combinations of two or more activities.

### Association between exercise habits and physical frailty

The participants with an exercise habit had a lower risk of physical pre-frailty + frailty compared to those without an exercise habit. However, compared to the participants who engaged in none of the activities, those with only an exercise habit were not at a lower risk of physical pre-frailty and frailty. It is already reported that exercise was significantly associated with a lower OR of longitudinal frailty onset in another study of community-dwelling older adults [24]. Zhang et al. observed a significant negative association between frailty and exercise in community-dwelling older adults [25]. Unlike our present study, the classification of exercise levels in the Zhang et al. study and other investigation was based on frequency only, and those studies did not consider the intensity of physical activity or social participation. The differences between the present study and those previous studies may be due to the influence of the small number of participants in the exercise habit-only group in our study (n = 60). Moreover, it is possible that exercise habits surveyed by a self-completed questionnaire may have overestimated the amount of exercise, the presence of low MVPA and light physical activity (activity intensity of 1.5 METs to < 3.0 METs), and low social involvement. These combined influences may not have contributed to the lower risk of physical pre-frailty and frailty among the participants with an exercise habit alone as defined in our study.

### Association between social participation and physical frailty

In the present study, compared to the participants without an exercise habit, social participation, or MVPA, those with only social participation were not at a lower risk of physical pre-frailty and frailty. An earlier investigation revealed that community-dwelling older adults who engaged in socializing with friends, hobby group activities, sports activities, and volunteer activities almost every day had a significantly lower risk of frailty at 4 years than those who did not engage in any of these activities [26]. Possible reasons for this difference compared to our present findings may be the influence of the small number of those with social participation alone in our study (n = 76) and/or the presence of a low level of social participation. Moreover, although the earlier study’s results were adjusted for physical activity, the results were assessed using the question of merely whether the participants engaged in ≥ 10 min of moderate-to-high intensity or light-energy-expenditure physical activity each week, and the influence of an exercise habit was not considered [26]. The participants’ physical activity was measured objectively by an accelerometer, and we also considered the influence of an exercise habit in our analyses. The results of the analyses thus clarified the impact of social activity alone as defined herein. Diversity in terms of exercise and the intensity of social participation may be necessary for combatting physical pre-frailty and frailty.

### Association between MVPA and physical frailty

Compared to our participants without an exercise habit, social participation, or MVPA, those with only MVPA (as defined in this study) had a lower risk of physical pre-frailty and frailty. Both an exercise habit and social participation contributed to a lower risk of physical pre-frailty and frailty if the MVPA criterion was met. It has been reported that in community-dwelling older adults, engagement in an accelerometer-recorded MVPA of less than 7.5 min/day was longitudinally related to a higher risk of developing physical frailty [27]. Several cross-sectional studies also reported a negative association between MVPA evaluated by an accelerometer and physical frailty among community-dwelling older adults [28–30]. None of the previous studies considered the effects of an exercise habit or social participation as was done in the present analyses. Even after accounting for the influence of an exercise habit and social participation, we observed that the participants with longer engagement in MVPA had a lower risk of physical pre-frailty and frailty compared to those with shorter engagement. MVPA was strongly associated with the low-activity component of the frailty components; this may be due to the frailty phenotype used in our study.

### Mechanisms regarding the association between Association between various type of activities and physical frailty

In older adults who engage in a composite implementation of the three activities defined in this study, the risks of physical pre-frailty and frailty were found to be low. Cheng et al. reported that in 163 community residents, upper-extremity muscle strength, flexibility, and chair-rise speed were cross-sectionally significantly higher in the residents who engaged in regular physical activity compared to those who were inactive [31]. Other studies of community-dwelling older adults demonstrated that a walking intervention improved depressive symptoms and that exercise increased the subjects’ insulin-like growth factor (IGF)-1 level and reduced inflammation [32, 33]. A 2014 review revealed that a high level of physical activity reduced the risk of cognitive dysfunction [34]. It has also been reported that community-dwelling older adults who participate in social activities have faster gait speeds [35], lower levels of psychological distress [36], higher cognitive function [37], lower inflammation, and higher IGF-1 values [38]. Moreover, Morie et al. showed that older men with higher levels of physical activity had higher physical function than those with lower levels [39]. Community-dwelling older adults with high MVPA were also reported to have lower depressive symptoms and higher IGF-1 [40, 41]. Other studies revealed that the amount of physical activity is positively associated with brain volume and brain functions such as executive function and memory, and negatively associated with inflammatory markers [42, 43]. Combined activity interventions such as various types of exercise and cognitive stimulation for patients with mild cognitive impairment have been reported to improve cognitive function and increase the volume of the hippocampus [44]. We thus speculate that our finding that any combination of exercise, social participation, and MVPA was associated with a lower risk of physical frailty is due to synergistic effects of these factors. The similar low risk of physical pre-frailty and frailty provided by any combination of two or more activities may be due to the balanced stimulation of the body by a variety of activities.

### Strengths of this study

The strengths of this study are as follows. Physical frailty was measured objectively and was based on criteria that considered body size and gender. Exercise habit was defined in detail in terms of frequency, time, and duration. The effects of exercise, social participation, and the intensity of physical activity were considered both alone and in combination. For these reasons, the results of this study may provide important public health information regarding the identification of physical activities involved in the effective prevention of physical frailty.

### Study limitations

The first limitation in this study is that the final-analysis subjects were able to walk on their own and completed the questionnaires with an interest in their own physical function. Further investigations are necessary to determine which approaches can be implemented to reduce the risk of physical frailty among individuals with low health awareness. Secondly, some of the participant groups in the eight categories had small n-values. With regard to the definition of activity, it should be noted that the results of this study depend on the definitions of exercise habits, social participation, and MVPA used. Studies with larger sample sizes are needed. Thirdly, we suspected that endogeneity of the physical frailty components low activity and MVPA was present. We thus conducted an additional analysis using four-item frailty (which excludes low-activity from the components of physical frailty) as the outcome; the existence of endogeneity was confirmed. However, this does not mean that MVPA is unassociated with a reduction of the risk of frailty. Besides, we revealed through this analysis that practicing a combination of two or more types of activity is effective in improving/maintaining frailty components other than physical activity. Fourth, in our logistic regression analyses in which stratified analysis and variable adjustment were conducted simultaneously, the resulting statistical models might have become excessively intricate, impeding the interpretation of the results. However, the results of our additional binomial logistic regression analysis excluding gender and BMI from the adjustment factors were essentially the same as that before this manipulation; the respective ORs (95%CI) for physical pre-frailty + frailty compared to none were 0.63 (0.32–1.25) for Exercise habit, 1.10 (0.56–2.17) for Social participation, 0.51 (0.29–0. 89) for MVPA, 0.44 (0.25–0.78) for Exercise habit + Social participation, 0.28 (0.16–0.48) for Exercise habit + MVPA, 0.28 (0.14–0.54), Social participation + MVPA, and 0.21 (0.12–0.36) for All activities, which means that this statistical problem made no essential impact on our results. Fifth, the causal relationships were not clarified, as this was a cross-sectional study. It is necessary to investigate causal relationships among physical activity, social interaction, and physical frailty longitudinally and determine whether the present results can help lead to the prevention of physical frailty.

## Conclusion

Among community-dwelling older Japanese adults, those who are engaged in MVPA or a combination of two or more types of activities from Exercise habit, Social participation, and MVPA have lower risks of physical pre-frailty or frailty and any of their components.

### Electronic supplementary material

Below is the link to the electronic supplementary material.

Supplementary Material 1

Supplementary Material 2

## Data Availability

The data used in this study are available from Hiro Kishimoto upon reasonable request and with permission. However, restrictions apply to the availability of these data, as they were used under license for this study and are not publicly available.

## Abbreviations

BMI: Body mass index
IGF: Insulin-like growth factor
MMSE: Mini-Mental State Examination
MPA: Moderate physical activity
MVPA: Moderate-to-vigorous physical activity
PAEE: Physical activity energy expenditure
VPA: Vigorous physical activity
