# Supplementary material for: Associations between various types of activity and physical frailty in older Japanese: a cross-sectional study
Source: BMC Geriatr. 2023 Nov 29;23:785. doi: 10.1186/s12877-023-04501-0 (PMC10685653; doi:10.1186/s12877-023-04501-0)
Supplement: Supplementary file 1 — Supplementary Material 1 [file 12877_2023_4501_MOESM1_ESM.docx]

**Additional File 1.** Associations with components of physical frailty for each activity combination

| **Group** | **n (%)** | **No. of applicable  persons (%)** | **Model 1** | | | **Model 2** | | |
| --- | --- | --- | --- | --- | --- | --- | --- | --- |
|  |  |  | **OR** | **95%CI** | **p-value** | **OR** | **95%CI** | **p-value** |
| **Weakness** | | | | | | | | |
| None | 139 (16.7) | 34 (24.5) | 1.00 | ref. | − | 1.00 | ref. | − |
| Exercise habit | 60 (7.2) | 16 (26.7) | 1.12 | 0.56–2.24 | 0.74 | 1.11 | 0.54–2.27 | 0.78 |
| Social participation | 76 (9.2) | 19 (25.0) | 1.03 | 0.54–1.97 | 0.93 | 1.09 | 0.56–2.12 | 0.81 |
| MVPA | 115 (13.8) | 26 (22.6) | 0.90 | 0.50–1.62 | 0.73 | 0.96 | 0.51–1.80 | 0.89 |
| Exercise habit + Social participation | 104 (12.5) | 18 (17.3) | 0.65 | 0.34–1.22 | 0.18 | 0.60 | 0.31–1.15 | 0.12 |
| Exercise habit + MVPA | 116 (14.0) | 18 (15.5) | 0.57 | 0.30–1.07 | 0.08 | 0.54 | 0.28–1.05 | 0.07 |
| Social participation + MVPA | 63 (7.6) | 12 (19.1) | 0.73 | 0.35–1.52 | 0.40 | 0.78 | 0.36–1.70 | 0.54 |
| All activities | 158 (19.0) | 28 (17.7) | 0.67 | 0.38–1.17 | 0.16 | 0.63 | 0.35–1.16 | 0.14 |
| **Slowness** | | | | | | | | |
| None | 139 (16.7) | 44 (31.7) | 1.00 | ref. | − | 1.00 | ref. | − |
| Exercise habit | 60 (7.2) | 16 (26.7) | 0.79 | 0.40–1.54 | 0.48 | 0.84 | 0.42–1.72 | 0.64 |
| Social participation | 76 (9.2) | 24 (31.6) | 1.00 | 0.55–1.82 | 0.99 | 1.04 | 0.55–1.95 | 0.91 |
| MVPA | 115 (13.8) | 26 (22.6) | 0.63 | 0.36–1.11 | 0.11 | 0.82 | 0.44–1.51 | 0.52 |
| Exercise habit + Social participation | 104 (12.5) | 19 (18.3) | 0.48 | 0.26–0.89 | 0.02 | 0.47 | 0.25–0.88 | 0.02 |
| Exercise habit + MVPA | 116 (14.0) | 12 (10.3) | 0.25 | 0.12–0.50 | <0.0001 | 0.30 | 0.14–0.61 | 0.001 |
| Social participation + MVPA | 63 (7.6) | 6 (9.5) | 0.23 | 0.09–0.57 | 0.0015 | 0.28 | 0.11–0.72 | 0.0083 |
| Al activities | 158 (19.0) | 16 (10.1) | 0.24 | 0.13–0.46 | <0.0001 | 0.29 | 0.15–0.57 | 0.0003 |
| **Exhaustion** | | | | | | | | |
| None | 139 (16.7) | 26 (18.7) | 1.00 | ref. | − | 1.00 | ref. | − |
| Exercise habit | 60 (7.2) | 5 (8.3) | 0.40 | 0.14–1.09 | 0.07 | 0.40 | 0.14–1.12 | 0.08 |
| Social participation | 76 (9.2) | 11 (14.5) | 0.74 | 0.34–1.59 | 0.43 | 0.66 | 0.29–1.47 | 0.3 |
| MVPA | 115 (13.8) | 19 (16.5) | 0.86 | 0.45–1.65 | 0.65 | 0.83 | 0.41–1.69 | 0.61 |
| Exercise habit + Social participation | 104 (12.5) | 11 (10.6) | 0.51 | 0.24–1.11 | 0.08 | 0.54 | 0.25–1.18 | 0.12 |
| Exercise habit + MVPA | 116 (14.0) | 12 (10.3) | 0.50 | 0.24–1.05 | 0.07 | 0.53 | 0.25–1.15 | 0.11 |
| Social participation + MVPA | 63 (7.6) | 10 (15.9) | 0.82 | 0.37–1.82 | 0.63 | 0.92 | 0.40–2.13 | 0.85 |
| All activities | 158 (19.0) | 11 (7.0) | 0.33 | 0.15–0.69 | 0.0032 | 0.32 | 0.15–0.69 | 0.004 |
| **Shrinking** | | | | | | | | |
| None | 139 (16.7) | 13 (9.4) | 1.00 | ref. | − | 1.00 | ref. | − |
| Exercise habit | 60 (7.2) | 6 (10.0) | 1.08 | 0.39–2.98 | 0.89 | 1.22 | 0.43–3.44 | 0.71 |
| Social participation | 76 (9.2) | 9 (11.8) | 1.30 | 0.53–3.20 | 0.57 | 1.30 | 0.52–3.24 | 0.58 |
| MVPA | 115 (13.8) | 16 (13.9) | 1.57 | 0.72–3.41 | 0.26 | 1.36 | 0.60–3.10 | 0.46 |
| Exercise habit + Social participation | 104 (12.5) | 9 (8.7) | 0.92 | 0.38–2.24 | 0.85 | 1.02 | 0.41–2.53 | 0.96 |
| Exercise habit + MVPA | 116 (14.0) | 10 (8.6) | 0.91 | 0.39–2.17 | 0.84 | 0.85 | 0.35–2.08 | 0.72 |
| Social participation + MVPA | 63 (7.6) | 3 (4.8) | 0.49 | 0.13–1.77 | 0.27 | 0.47 | 0.12–1.75 | 0.26 |
| All activities | 158 (19.0) | 12 (7.6) | 0.80 | 0.35–1.81 | 0.59 | 0.71 | 0.30–1.66 | 0.43 |
| **Low physical activity** | | | | | | | | |
| None | 139 (16.7) | 51 (36.7) | 1.00 | ref. | − | 1.00 | ref. | − |
| Exercise habit | 60 (7.2) | 23 (38.3) | 1.07 | 0.57–2.00 | 0.83 | 1.22 | 0.59–2.53 | 0.59 |
| Social participation | 76 (9.2) | 35 (46.1) | 1.47 | 0.84–2.60 | 0.18 | 1.70 | 0.89–3.23 | 0.11 |
| MVPA | 115 (13.8) | 1 (0.9) | 0.02 | 0.002–0.11 | <0.0001 | 0.02 | 0.003–0.17 | 0.0003 |
| Exercise habit + Social participation | 104 (12.5) | 32 (30.8) | 0.77 | 0.45–1.32 | 0.34 | 0.70 | 0.38–1.31 | 0.26 |
| Exercise habit + MVPA | 116 (14.0) | 5 (4.3) | 0.08 | 0.03–0.20 | <0.0001 | 0.09 | 0.03–0.26 | <0.0001 |
| Social participation + MVPA | 63 (7.6) | 2 (3.2) | 0.06 | 0.01–0.24 | 0.0001 | 0.06 | 0.01–0.29 | 0.0003 |
| All activities | 158 (19.0) | 3 (1.9) | 0.03 | 0.01–0.11 | <0.0001 | 0.05 | 0.01–0.18 | <0.0001 |

Model 1: No adjustment factors. Model 2: Adjusted for age, gender, BMI, no. of diseases, no. of pain site, score of MMSE, smoking habit, alcohol habit, years of education, sedentary time. MVPA: moderate-to-vigorous physical activity.
