# Supplementary material for: Associations between various types of activity and physical frailty in older Japanese: a cross-sectional study
Source: BMC Geriatr. 2023 Nov 29;23:785. doi: 10.1186/s12877-023-04501-0 (PMC10685653; doi:10.1186/s12877-023-04501-0)
Supplement: Supplementary file 2 — Supplementary Material 2 [file 12877_2023_4501_MOESM2_ESM.docx]

**Additional File 2.** Association with physical pre-frailty or physical frailty when low physical activity is excluded in each combination

| **Group** | **n (%)** | **No. of four-item pre-frailty  + four-item frailty,**  **n (%)** | **Model 1** | | | **Model 2** | | |
| --- | --- | --- | --- | --- | --- | --- | --- | --- |
|  |  |  | **OR** | **95%CI** | **p-value** | **OR** | **95%CI** | **p-value** |
| None | 139 (16.7) | 82 (59.0) | 1.00 | ref. | − | 1.00 | ref. | − |
| Exercise habit | 60 (7.2) | 29 (48.3) | 0.65 | 0.35–1.20 | 0.17 | 0.65 | 0.35–1.21 | 0.18 |
| Social participation | 76 (9.2) | 47 (61.8) | 1.13 | 0.64–2.00 | 0.68 | 0.99 | 0.56–1.76 | 0.97 |
| MVPA | 115 (13.8) | 65 (56.5) | 0.90 | 0.55–1.49 | 0.69 | 0.88 | 0.52–1.49 | 0.63 |
| Exercise habit + Social participation | 104 (12.5) | 42 (40.4) | 0.47 | 0.28–0.79 | 0.0043 | 0.42 | 0.25–0.72 | 0.0016 |
| Exercise habit + MVPA | 116 (14.0) | 47 (40.5) | 0.47 | 0.28–0.78 | 0.0035 | 0.43 | 0.26–0.73 | 0.0017 |
| Social participation + MVPA | 63 (7.6) | 25 (39.7) | 0.46 | 0.25–0.84 | 0.01 | 0.49 | 0.26–0.92 | 0.03 |
| All activities | 158 (19.0) | 59 (37.3) | 0.41 | 0.26–0.66 | 0.0002 | 0.36 | 0.22–0.60 | <0.0001 |

Model 1: No adjustment factors. Model 2: Adjusted for age, gender, BMI, no. of diseases, no. of pain site, score of MMSE, smoking habit, alcohol habit, years of education, sedentary time. Four-item frailty and physical frailty were defined by four components of physical frailty (with low activity removed). Older adults who met three or more of the four-item frailty constructs were defined as being frail. Four-item pre-frailty: Older adults who fall under one or two were defined as having four-item pre-frailty.
